# Melkersson-Rosenthal Syndrome in a 38-Year-Old Female Patient: A Rare Case With Complete Triad

**DOI:** 10.7759/cureus.27427

**Published:** 2022-07-28

**Authors:** Georges Aoun, Nadia Skandri, Hafiza Ghattas, Carlo Maksoud

**Affiliations:** 1 Oral Medicine and Maxillofacial Radiology, Lebanese University, Beirut, LBN

**Keywords:** corticosteroid, fissured tongue, facial palsy, orofacial swelling, melkersson-rosenthal syndrome

## Abstract

Melkersson-Rosenthal syndrome (MRS) is an uncommon neuro-mucocutaneous disease, clinically characterized by a triad of recurrent facial palsy, orofacial swelling, and fissured tongue. This report presents the case of a 38-year-old female diagnosed with MRS based on its three clinical features. A corticosteroid (1 mg/kg/day of oral prednisolone) was prescribed for a week, and then tapered off over two weeks by gradually lowering the dose. Regular annual long-term follow-ups were requested to monitor the disease activity.

## Introduction

Melkersson-Rosenthal syndrome (MRS) is an uncommon neuro-mucocutaneous disease, clinically characterized by recurrent orofacial swelling (edema), relapsing facial palsy, and a fissured tongue [1]. The complete form of the disease including the three stated symptoms is very rare; most affected patients present only one or two symptoms [2]. MRS is reported in 0.08% of the general population, with a female to male ratio of 2:1. Most of the diagnosed cases occurred in people aged between 25 and 40 [3].

MRS etiology remains unknown, although many studies have supposed that genetic, allergic, infectious, and immunologic issues can possibly be contributing factors to its pathogenesis [4]. There is no specific treatment for MRS; symptoms management consists of anti-inflammatory agents, antihistamines, corticosteroids and in some cases surgery [3]. Herein we present a rare case of MRS in a 38-year-old female patient.

## Case presentation

**Figure 1:**
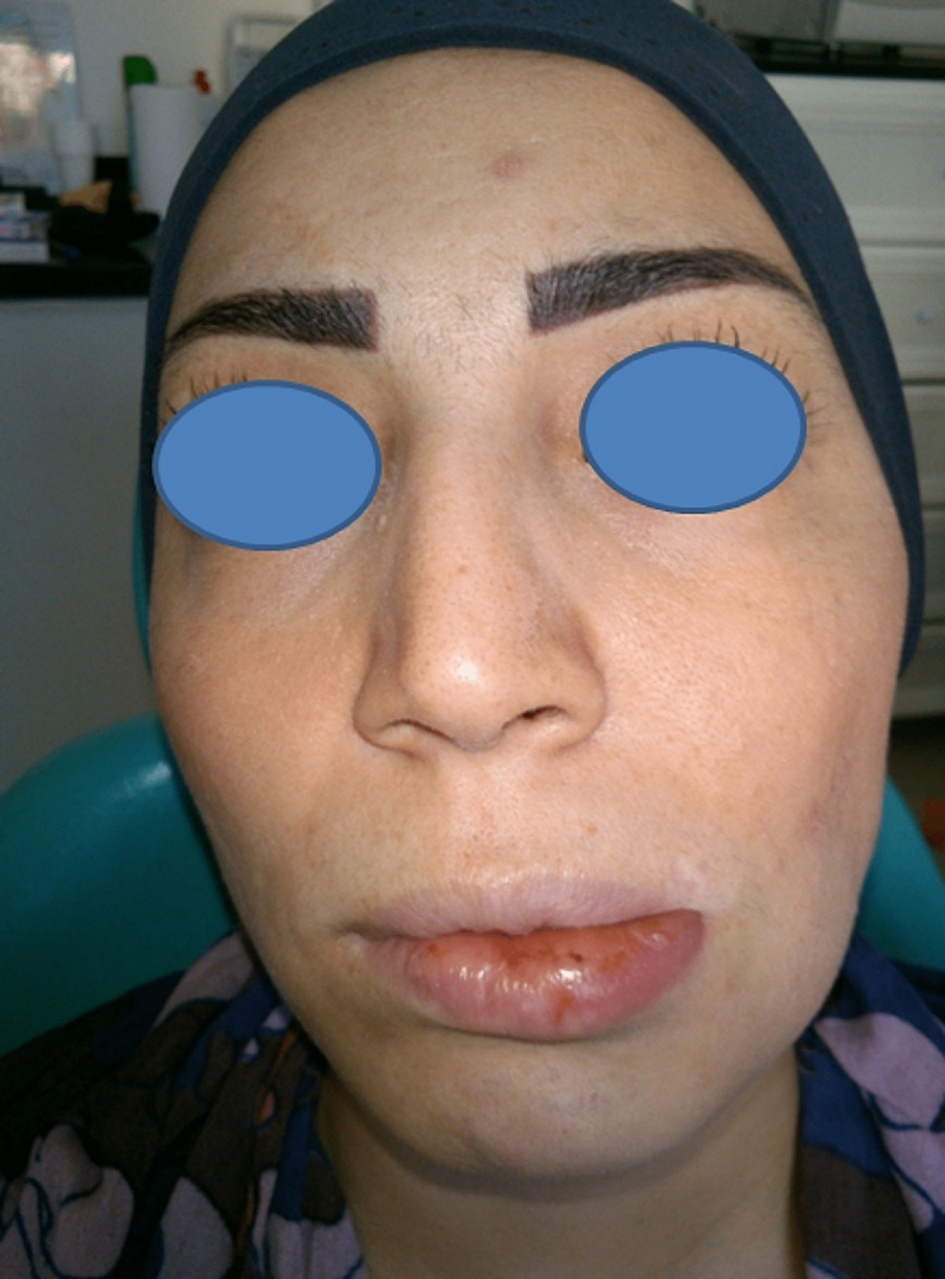
Photograph showing the swelling of the lower lip

A 38-year-old female was referred by her dentist to our Department of Oral Medicine and Maxillofacial Radiology for evaluation of recurrent swelling of the lower lip along with left hemifacial palsy (Figure 1).

**Figure 2:**
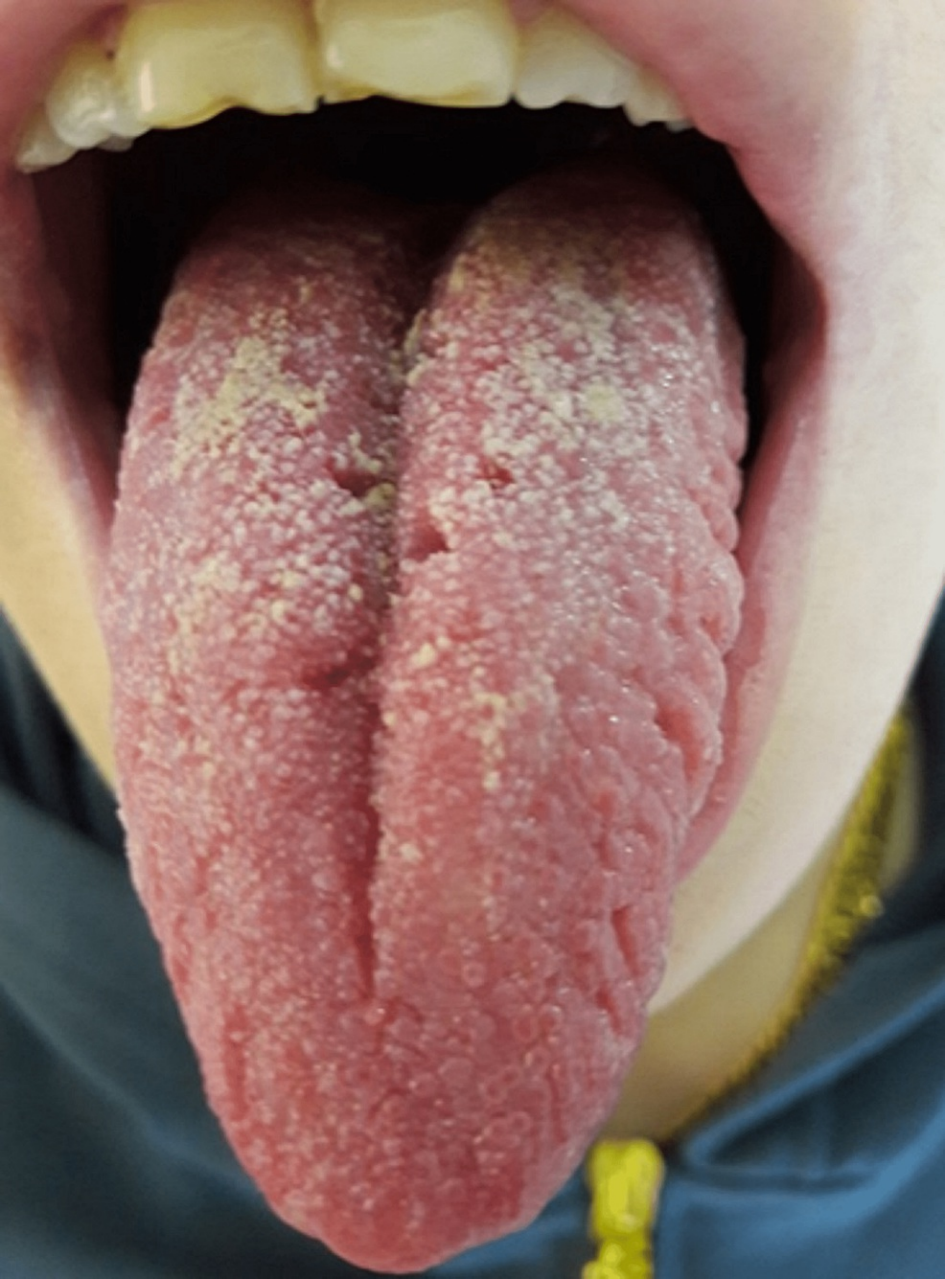
Photograph showing the fissured tongue

Her medical record revealed that these two symptoms occurred once a year since she was 28 years old; most of the time, previous episodes resolved in a few days without treatment. She has a history of smoking (10 cigarettes per day) for more than 18 years. Her physical examination disclosed no extra-oral abnormalities. Intraorally, the patient presented a fissured coated tongue (Figure 2).

**Figure 3:**
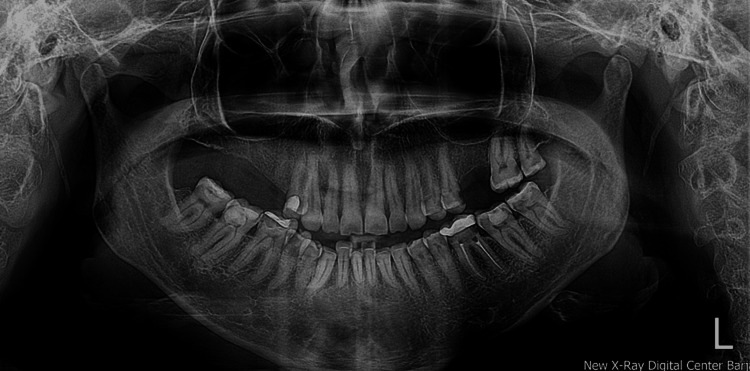
Digital panoramic radiograph of the patient

A panoramic radiograph was requested to rule out any unusual findings in the teeth and surrounding bone structures. The radiological result was unremarkable (Figure 3).

Based on the clinical presentation (recurrent swelling of the lower lip, left hemifacial palsy, and fissured tongue) a diagnosis of MRS was given. Corticosteroid (50 mg/day, oral prednisolone) was prescribed for a week, and then tapered off over two weeks by gradually lowering the dose. Regular annual long-term follow-ups were requested to monitor the disease activity.

## Discussion

MRS is a syndrome characterized by a triad of clinical symptoms [5]. The term oligosymptomatic MRS is used when two of these clinical features are present, and monosymptomatic MRS is used in case of one [3]. Histopathological evidence of epithelioid non-caseous granulomas is needed to confirm the monosymptomatic MRS diagnoses [3,6]. All three features do not often co-occur (8-18% of all patients) [7]. Facial palsy, which can be either unilateral or bilateral, occurs in 30-90% of cases. It is generally the first clinical feature preceding orofacial swelling by months to years. Its duration increases with the progression of the disease [3]. As for fissured tongue, it is found in 30-80% of cases of MRS [8]. On the other hand, MRS swelling, which is the most common and major feature of MRS, may be the only symptom occurring. It is painless, non-pitting and may be seen in various facial regions such as the lips, one or both cheeks, eyelids, or rarely, one side of the scalp [7-9]. In the first occurrence, swelling may resolve in a few hours or days, but in subsequent episodes, it may last longer and in some cases be able to become permanent. The swollen lips may be painful and appear cracked and discolored. Partial or total non-resolution of swelling may lead to fibrosis and consequently permanent disfiguration of the face [3].

Besides these three basic symptoms, patients with MRS have also reported migraine, headache, dizziness, xerostomia, and other neurological symptoms such as tinnitus, sudden deafness, dysphagia, hypogeusia, reduced or excessive facial sweating, excessive eye tears, and visual disturbances [3,6]. Non-neurological symptoms of MRS may also include diverticulitis and uveitis. Other cranial nerves including trigeminal, olfactory, auditory, glossopharyngeal, and hypoglossal nerves can also be involved [10]. MRS recurrence may lead to personality changes, anxiety, and depression [3].

MRS etiology is not clear. Its etiopathogenesis has been suggested to be associated with many other conditions such as viral infections (herpes simplex virus-HSV1), mycobacterial infections (tuberculosis, leprosy), hereditary granulomatous disorders, Down’s syndrome, psoriasis, thyroiditis, multiple sclerosis, keratitis, diabetes mellitus, sarcoidosis, Crohn’s disease, ulcerative colitis, and allergic disorders [8,9,11]. However, no strong points exist to explain the etiopathogenesis of MRS. Additionally, there is evidence to suggest that MRS may be inherited because familial inheritance was observed in a number of patients. Nevertheless, the presentation of symptoms is extremely unpredictable, which makes diagnosis and observing inheritance a challenge.

There is no regular treatment for MRS. Most signs and symptoms of the disease resolve spontaneously. Yet, in some patients the condition may be progressive, leading to facial disfiguring and amplifying residual paralysis [12]. To prevent these complications, corticosteroids were traditionally prescribed. There is evidence to believe that corticosteroids lead to 50-80% of cases improvement and reduce relapse occurrence by 60-75% [9]. Brief courses of immunosuppressants, non-steroidal anti-inflammatory drugs, antihistamines, and antibiotics were also prescribed depending on the case [3]. Surgery is occasionally recommended to reduce swollen lips or to decompress affected facial nerves [12]. In our case, oral prednisolone proved to be useful, especially in improving facial palsy.

## Conclusions

MRS is a rare disorder, difficult to diagnose and treat. Its most frequent symptom is the recurrent swelling in the orofacial area. Dental practitioners should keep MRS in mind for the patients who present with lip swelling and they should automatically refer the patients in question for specialized medical evaluation.
